# Integrated Transcriptome and Metabolome Analysis Reveals Differential Berberine Biosynthesis in Leaves and Stems of *Phellodendron amurense* Rupr. Plantlets

**DOI:** 10.3390/cimb48050464

**Published:** 2026-04-29

**Authors:** Wei Li, Xuefang Wang, Cancan Lv, Yiqing Wang, Yan Wang, Tuuli-Marjaana Koski, Kang Du, Jun Wang

**Affiliations:** 1 State Key Laboratory of Tree Genetics and Breeding, Beijing Forestry University, Beijing 100083, China; 2 Experimental Center of Desert Forestry, Chinese Academy of Forestry, Bayannur 015200, China; 3 School of Biological Sciences and Technology, Beijing Forestry University, Beijing 100083, China; 4 The National Facility Preservation Bank for Forestry and Grassland Germplasm Resources (Xiong’an), Beijing Forestry University, Beijing 100083, China; 5 Forestry and Grassland Research Institute of Tongliao City, Tongliao 028000, China

**Keywords:** berberine, leaf, metabolome, *Phellodendron amurense*, stem, transcriptome

## Abstract

*Phellodendron amurense* Rupr. is a native tree species in China, well known for its significant medicinal value. Its pharmacological activity mainly derives from the abundant isoquinoline alkaloids in its bark. Berberine serves as the key compound underlying the multiple pharmacological effects of *P. amurense* and exhibits organ-specific accumulation. However, the genetic mechanisms governing this organ-specific accumulation remain unclear. Genes encoding O-methyltransferase (OMT) and cytochrome P450 (CYP) may play an important role in this regulatory process. In this study, by integrating transcriptomic and metabolomic data from the leaves and stems of *P. amurense* plantlets, we identified core candidate genes and transcription factors (TFs) that regulate the differential biosynthesis of berberine between these two organs. The results showed that 37 metabolites were significantly upregulated in stems, including main medicinal components such as berberine and jatrorrhizine, while 8497 genes were differentially expressed between leaves and stems. Among these, downstream genes in the berberine biosynthesis pathway, including *OMT*s and *CYP*s, were predominantly highly expressed in stems. A co-expression regulatory network identified some TFs such as *PaBES1*, *PaWRKY12*/*13*, *PaNAC5*, and *PaMYB12* as the key nodes regulating the differential biosynthesis of berberine. Phylogenetic analysis classified the 97 *PaOMT*s into four subgroups. Core candidate genes such as *PaOMT7* and *PaOMT9* were contained in subgroup IV, potentially contributing to the specific modification of characteristic alkaloids in *P. amurense*. This study reveals the transcriptional regulatory networks underlying the organ-specific accumulation of berberine in *P. amurense* plantlets, providing key targets and theoretical support for the targeted improvement and development of elite medicinal varieties.

## 1. Introduction

*Phellodendron amurense* Rupr. is a deciduous tree species from the genus *Phellodendron* in the family Rutaceae. It serves as the unique botanical source of Chinese Corktree Bark, possessing various pharmacological activities, including antibacterial, anti-inflammatory, hypoglycemic, and antitumor effects [1]. Benzylisoquinoline alkaloids (BIAs), including magnoflorine, berberine, and palmatine, etc., are a major class of bioactive ingredients in the therapeutic efficacy of *P. amurense* and hold significant medicinal potential [1,2]. Recently, the key enzymes involved in the pathway of BIA biosynthesis have been largely elucidated. For instance, 6-O-methyltransferase (6OMT) and scoulerine 9-O-methyltransferase (SOMT) from *Coptis chinensis* Franch. (Ranunculaceae), as well as berberine bridge enzyme (BBE), SOMT, and 7-O-methyltransferase (7OMT) from *Corydalis tomentella* Franch. (Papaveraceae), all participate in BIA synthesis [3,4].

Berberine, belonging to BIA, is widely distributed in families such as Rutaceae, Ranunculaceae, Papaveraceae, Berberidaceae, and Menispermaceae [5]. It is the key compound underlying the pharmacological effects of *P. amurense*. Previous studies have reported that berberine exhibits broad-spectrum antibacterial activity by inhibiting the activity of filamentous temperature-sensitive mutant Z (FtsZ), disrupting the integrity of the cell wall and membrane, and interfering with DNA and RNA synthesis [6]. Furthermore, berberine could stimulate insulin secretion [7] and enhance glucose metabolism by stimulating glycolysis [8]. In addition, berberine effectively inhibits tumorigenesis by suppressing proliferative kinase signaling and arresting the cell cycle [9]. These multifaceted pharmacological effects contribute to the high medicinal value of *P. amurense*. Additionally, berberine also plays multifaceted roles in plants, including enhancing antimicrobial defense and improving resistance against viruses and insects, among others [10,11]. However, due to the scarcity of its wild resources, *P. amurense* was listed as a National Key Protected Wild Plants of China in 2021 [12]. It is urgent to efficiently increase the content of effective medicinal compounds in *P. amurense* through metabolic engineering and molecular breeding.

Recently, six key enzymatic steps in the process of berberine biosynthesis in *P. amurense* have been clarified, revealing that an O-methyltransferase (OMT) possesses N-methylation activity, and the *CYP71BG29* that encodes cytochrome P450 (CYP) functions as a BBE in this process [13]. These findings provide new insights into the enzymatic mechanisms underlying berberine biosynthesis. Notably, the content of medicinal components often varies across different organs within the same individual of *P. amurense* [14]; for instance, berberine and jatrorrhizine are significantly more abundant in the bark than in perennial branches, annual branches, and leaves of natural wild trees. However, the genetic mechanisms governing this organ-specific accumulation of BIAs in *P. amurense*, particularly berberine, remain unclear.

Furthermore, previous studies about *P. amurense* have primarily focused on the BIA biosynthesis in trees growing outdoors [2,15]. The culture system of plantlets offers advantages such as high environmental controllability and the potential for optimizing secondary metabolism, making it an ideal platform for exploring the genetic mechanisms of secondary metabolism in medicinal plants and efficiently producing bioactive compounds. In this study, by integrating transcriptomic and metabolomic data from leaves and stems of *P. amurense* plantlets, we identified differences in the accumulation of various secondary metabolites, particularly berberine, between these two organs. The core candidate genes involved in the differential biosynthesis of berberine were also systematically revealed. This study aimed to provide theoretical support and genetic targets for the metabolic engineering and the development of germplasm with enhanced medicinal value of *P. amurense*.

## 2. Materials and Methods

### 2.1. Plant Materials and Growth Conditions

The clone Pa-19 of *P. amurense* was used as the experimental material. Aseptic plantlets of this clone were cultured on solid 1/2 MS medium (Solarbio, Beijing, China) supplemented with 3.0 mg·L^−1^ indole-3-butyric acid (IBA, Solarbio, Beijing, China), 0.1 mg·L^−1^ 1-naphthaleneacetic acid (NAA, Caisson Labs, Logan, UT, USA), 30 g·L^−1^ sucrose (Biorigin, Beijing, China), and 6 g·L^−1^ agar (Biorigin, Beijing, China). The culture conditions were maintained at 25 ± 1 °C under cool-white fluorescent light (30–40 µmol·m^−2^·s^−1^) with a photoperiod of 16 h light/8 h dark. The fourth to sixth leaves and the corresponding stems of healthy 60-day-old plantlets were collected. Three biological replicates, each consisting of five pooled plantlets, were performed, and the leaf and stem samples were designated as Leaf-1, Leaf-2, Leaf-3 and Stem-1, Stem-2, Stem-3, respectively. All samples were stored at −80 °C for subsequent analyses.

### 2.2. Determination of Alkaloid Contents and Identification of Differentially Accumulated Alkaloids

Frozen samples were freeze-dried under vacuum and ground using a mixer mill (MM400, Retsch, Shanghai, China) with a zirconia bead at 30 Hz for 1.5 min. The following procedures were performed following the method described by Li et al. [16]. In brief, approximately 50 mg of lyophilized powder from each sample was mixed with 1.2 mL of pre-cooled (4 °C) 70% aqueous methanol (Merck KGaA, Darmstadt, Germany). The mixture was vortexed for 30 s every 30 min for a total of six times, then thoroughly mixed and centrifuged at 12,000 rpm for 3 min. The supernatant was filtered through a 0.22 µm organic nylon membrane (SCAA-104, ANPEL, Shanghai, China) for subsequent metabolite analysis. Subsequently, the filtered samples were used for UPLC–MS/MS analysis by a UPLC–ESI–MS/MS system (ExionLC™ AD UPLC and 4500 Q TRAP mass spectrometer, both from SCIEX, Framingham, MA, USA) at MetWare Biotechnology Co., Ltd., Wuhan, China.

To verify the reproducibility among samples, principal component analysis (PCA) and orthogonal partial least squares discriminant analysis (OPLS-DA) were performed using R v4.5.3. Differentially accumulated alkaloids (DAAs) were screened based on variable importance in projection (VIP) ≥ 1 and fold change ≥ 2 or ≤0.5. The identified metabolites were annotated using the Kyoto Encyclopedia of Genes and Genomes (KEGG) compound database (http://www.kegg.jp/kegg/compound/, accessed on 1 December 2025) and subjected to metabolite set enrichment analysis (MSEA).

### 2.3. Transcriptomic Analysis

Total RNA was extracted from both stems and leaves, each with three biological replicates, resulting in six RNA-seq libraries. Sequencing was performed on the Illumina NovaSeq 6000 platform to generate paired-end reads. The genome of *P. amurense* assembled by Xu et al. [13] was used as the reference genome, and the reference genome index was constructed using HISAT2 v2.2.1 [17], incorporating exon and splice site information extracted from the gene annotation file (converted to GTF format via GffRead v0.12.7 [18]). Paired-end reads were aligned to the indexed genome using HISAT2 to retain information relevant for transcriptome reconstruction. Alignment files (SAM) were converted to BAM format, sorted, and indexed using SAMtools v1.23 [19]. Gene-level quantification was conducted with featureCounts v2.0.4 [20] using the original annotation, and count matrices from all samples were combined for downstream analysis. Raw counts were normalized to transcripts per million (TPM) values to facilitate cross-sample comparison of gene expression levels.

Differential expression analysis between stems and leaves was performed using the R package DESeq2 v1.50.2 [21]. Genes with |log_2_Fold Change| > 1 and false discovery rate (FDR) < 0.05 were defined as differentially expressed genes (DEGs). To investigate the biological significance of the DEGs, GO and KEGG enrichment analyses were conducted using the R package clusterProfiler v4.0 [22,23], with an adjusted *p*-value < 0.05 as the significance threshold.

### 2.4. Validation by Quantitative Real-Time PCR

Total RNA (1 μg) extracted previously was reverse transcribed into cDNA using the HiScript II Q RT SuperMix for qPCR (+gDNA wiper) kit (R223-01, Vazyme, Nanjing, China) according to the manufacturer’s instructions. Quantitative real-time PCR (RT-qPCR) was performed on an Applied Biosystems QuantStudio 6 Flex real-time PCR system (Thermo Fisher, Waltham, MA, USA) using ChamQ Universal SYBR qPCR Master Mix (Q711-02, Vazyme, Nanjing, China). The 20 μL reaction mixture consisted of 10 μL of SYBR Master Mix, 5 μL of diluted cDNA, 1 μL each of forward and reverse primers (10 μM), and 3 μL of nuclease-free water. The thermal cycling conditions were as follows: initial denaturation at 95 °C for 2 min, followed by 40 cycles of 95 °C for 15 s, 55 °C for 30 s, and 72 °C for 10 s. Melting curve analysis was performed at the end of each run to verify amplification specificity. The *18S rRNA* gene was used as the endogenous reference for normalization. All primers were designed using SnapGene v8.2.2 (https://www.snapgene.com/, accessed on 8 December 2025), and the sequences are listed in Table S1. Relative expression levels of target genes were calculated using the 2^−ΔΔCt^ method [24].

### 2.5. Identification of Key Genes

According to the key steps in BIAs biosynthesis defined by Xu et al. [13], the key DEGs involved in berberine and BIAs biosynthesis in *P. amurense* were identified. Their promoter sequences were extracted using TBtools v1.120 [25], and cis-acting elements were predicted using the PlantCARE database (https://bioinformatics.psb.ugent.be/webtools/plantcare/html/, accessed on 15 December 2025) to infer the regulatory relationships between key genes and transcription factors (TFs). A co-expression network was visualized using Cytoscape v3.10.1 [26].

### 2.6. Construction of Phylogenetic Tree

To construct the phylogenetic tree, ten *OMT*s from *P. amurense* and six *OMT*s from *Arabidopsis thaliana* (L.) Heynh. were aligned using MEGA v7.0 [27]. The tree was constructed using the neighbor-joining method with 1000 bootstrap replicates. The resulting tree was then visualized and refined using the online tool EvolView v3 (https://www.evolgenius.info/evolview/#/, accessed on 6 January 2026).

## 3. Results

### 3.1. Differential Accumulation of Alkaloids in Leaves and Stems

**Figure 1:**
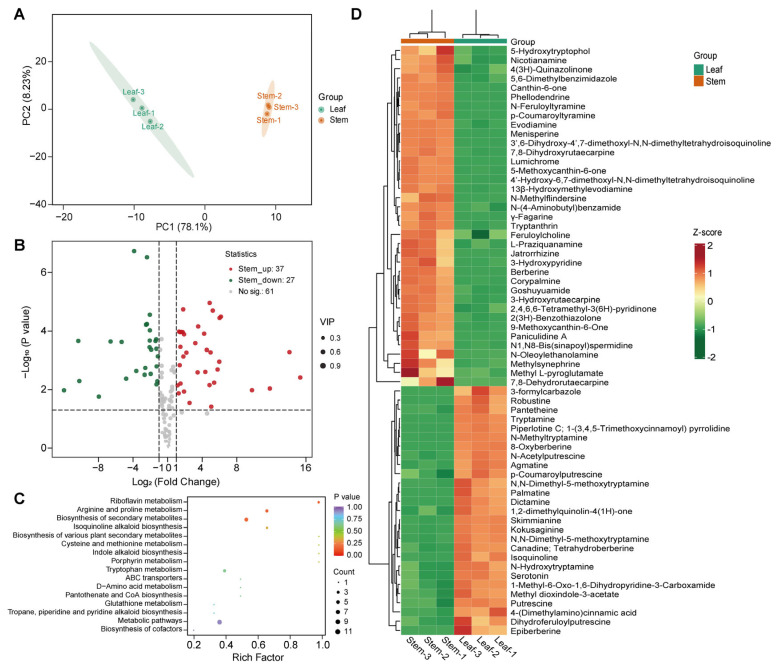
Differential accumulation of metabolites in leaves and stems of *Phellodendron amurense*. (**A**) principal component analysis (PCA) of metabolites in leaves and stems; (**B**) volcano plot of differentially accumulated alkaloids (DAAs) between leaves and stems. The vertical dashed lines mark the |log_2_ (Fold Change)| = 1 threshold, and the horizontal dashed line indicates the significance threshold of *p* = 0.05; (**C**) KEGG enrichment analysis of DAAs between leaves and stems; (**D**) clustered heatmap showing the expression levels of DAAs in leaves and stems.

PCA showed that the metabolite profiles of *P. amurense* leaves and stems were clearly distinct, with high reproducibility among biological replicates, indicating reliable metabolomic data (Figure 1A). Differential metabolite analysis identified 64 DAAs between leaves and stems, of which 37 were upregulated and 27 were downregulated in stems (Figure 1B). KEGG annotation revealed that 11 and 7 DAAs were enriched in metabolic pathways (ko01100) and biosynthesis of secondary metabolites (ko01110), respectively, while 2 DAAs were directly enriched in isoquinoline alkaloid biosynthesis (ko00950) (Figure 1C). Notably, isoquinoline (pmb0785) was upregulated in leaves, whereas several important BIAs in *P. amurense*, including phellodendrine (MWSmce360), berberine (MWSmce091), and jatrorrhizine (Hmdp005429), were significantly upregulated in stems (Figure 1D). These findings indicate a great accumulation of alkaloids in the stems of *P. amurense*.

### 3.2. Differential Expression of Genes in Leaves and Stems

**Figure 2:**
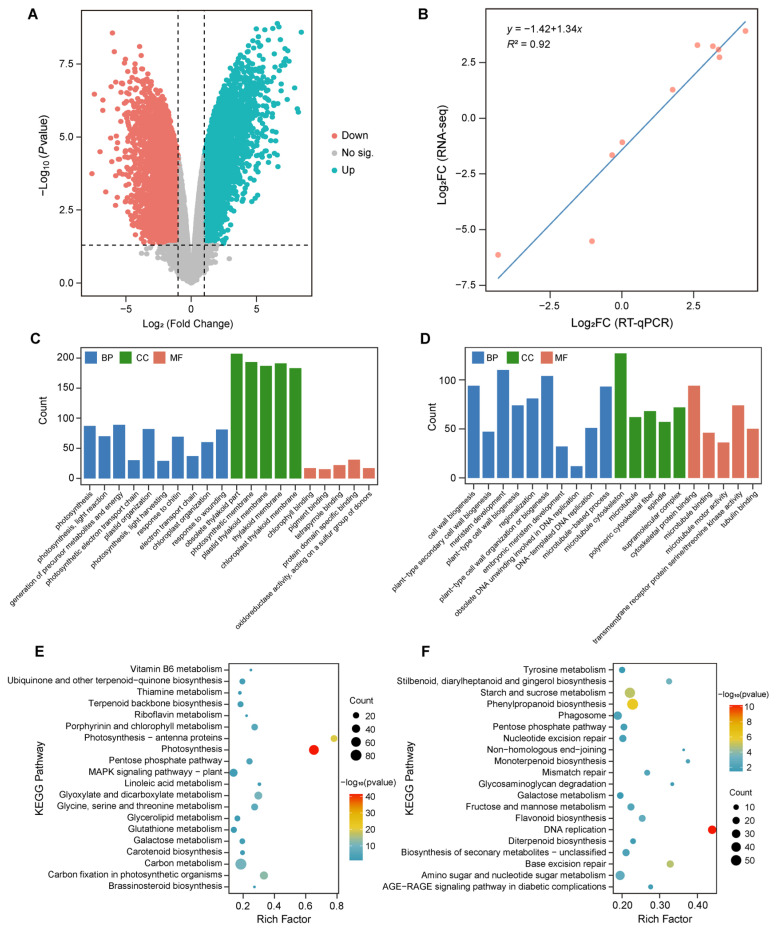
Differential expression of genes in leaves and stems of *Phellodendron amurense*. (**A**) volcano plot of expressed genes in leaves and stems. “Down” and “Up” indicate differentially expressed genes (DEGs) that downregulated and upregulated in stems, respectively, while “No sig.” indicates genes with no significant difference in expression levels between leaves and stems. The horizontal dashed line represents the significance threshold of *p* = 0.05, and the two vertical dashed lines mark the threshold of |log_2_ (Fold Change)| = 1; (**B**) correlation analysis between RT-qPCR results and RNA-seq data; (**C**,**D**) GO enrichment analysis of downregulated (**C**) and upregulated (**D**) DEGs in stems. BP, CC, and MF represent the three GO categories: biological process, cellular component, and molecular function, respectively; (**E**,**F**) KEGG enrichment analysis of downregulated (**E**) and upregulated (**F**) DEGs in stems.

A total of 8497 DEGs were identified between leaves and stems. Among these, 3139 DEGs were highly expressed in leaves, while 5358 DEGs showed higher expression in stems (Figure 2A). GO enrichment analysis indicated that genes highly expressed in leaves were primarily involved in biological processes such as photosynthesis (GO:0015979), generation of precursor metabolites and energy (GO:0006091), and plastid organization (GO:0009657), with their products mainly localized to the thylakoid membrane (GO:0042651) (Figure 2C). Consistently, these DEGs were significantly enriched in KEGG pathways related to photosynthesis, including photosynthesis (ko00195), glyoxylate and dicarboxylate metabolism (ko00630), and porphyrin and chlorophyll metabolism (ko00860) (Figure 2E). In contrast, genes highly expressed in stems were predominantly associated with biological processes such as cell wall biogenesis (GO:0042546) and microtubule-based processes (GO:0009834) (Figure 2D). Notably, 57 and 18 DEGs were significantly enriched in the phenylpropanoid biosynthesis (ko00940) and flavonoid biosynthesis (ko00941) pathways, respectively (Figure 2F), highlighting the transcriptional advantage of secondary metabolite synthesis in stems. The result of RT-qPCR showed strong consistency with the RNA-seq data (*R*^2^ = 0.92) (Figure 2B, Table S2), confirming the reliability of the transcriptomic analysis.

### 3.3. Differential Expression of Genes in the Key Pathway

**Figure 3:**
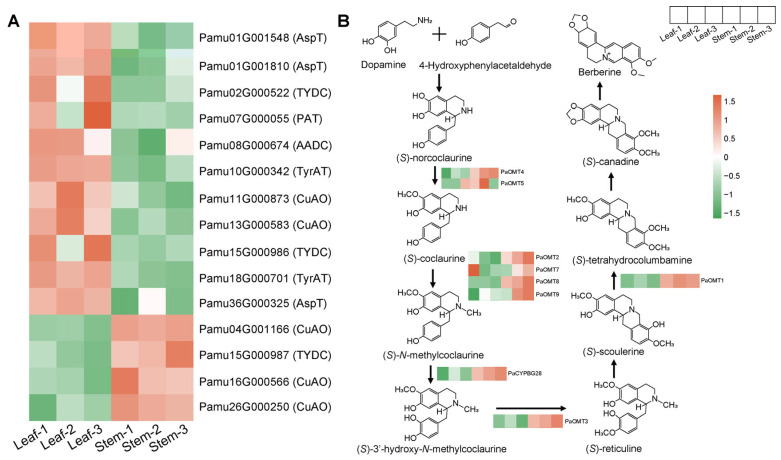
Differential expression of genes involved in the benzylisoquinoline alkaloid (BIA) and berberine biosynthesis pathways in leaves and stems of *Phellodendron amurense*. (**A**) heatmap showing the expression levels of genes involved in the BIA biosynthetic pathway; (**B**) expression differences in genes involved in the berberine biosynthetic pathway. The arrows indicate the direction of product synthesis.

The expression levels of DEGs involved in the BIA and berberine biosynthesis pathways in *P. amurense* leaves and stems were visualized (Figure 3). Several important genes in the BIA biosynthesis pathway, including Pamu01G001548 (aspartate aminotransferase, *AspT*), Pamu11G000873 (copper-containing amine oxidase, *CuAO*), and Pamu15G000986 (tyrosine/dopa decarboxylase, *TYDC*/*DDC*), were significantly downregulated in stems. In contrast, four genes, comprising three *PaCuAO*s (Pamu04G001166, Pamu16G000566, and Pamu26G000250) and one *PaTYDC* (Pamu15G000987), were significantly upregulated in stems (Figure 3A). In the berberine biosynthesis pathway, *PaOMT*s and *PaCYP*s play key roles. Among these, Pamu08G000115 (*PaOMT1*), Pamu06G000283 (*PaOMT2*), Pamu20G000479 (*PaOMT3*), and Pamu18G000966 (*PaCYP71BG28*) exhibited higher expression levels in stems than in leaves (Figure 3B). These genes may play important roles in regulating the differential biosynthesis of berberine.

### 3.4. Regulatory Network of Berberine Biosynthesis

**Figure 4:**
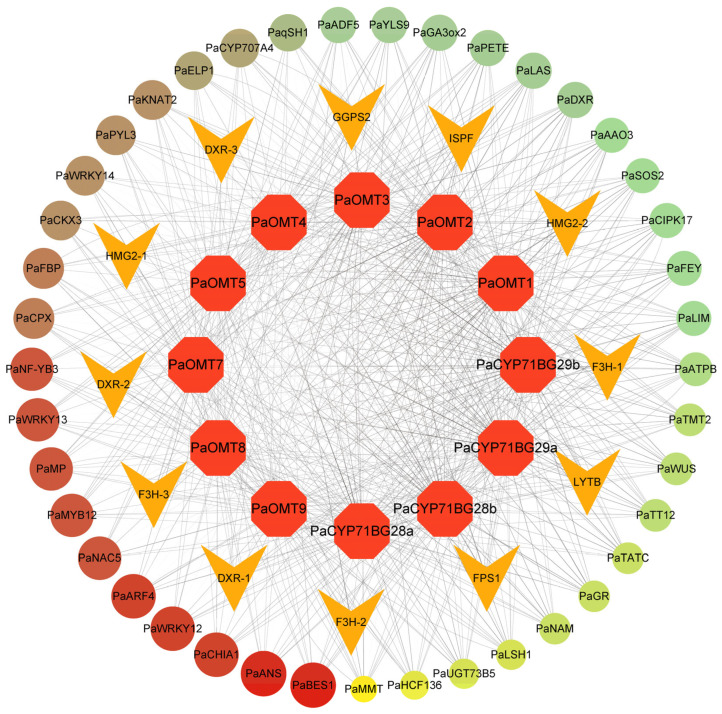
Regulatory network of berberine biosynthesis. Quadrilaterals and hexagons represent genes in the pathway of flavonoid biosynthesis and berberine biosynthesis, respectively, and circles represent transcription factors. The higher connectivity of transcription factors is indicated by the larger sizes and darker colors of circles.

A regulatory network was constructed based on candidate genes involved in the berberine biosynthesis (Figure 4). In this network, *OMT*s and *CYP*s were highly correlated with those of genes in the flavonoid biosynthesis pathway, such as *F3H*, *HMG2*, and *DXR*. They were broadly regulated by TFs from the WRKY, MYB, and NAC families. Notably, *PaBES1* (Pamu02G000366) exhibited the most regulatory relationships, underscoring its central role in the network governing berberine biosynthesis. Additionally, *PaWRKY12* (Pamu14G000149), *PaWRKY13* (Pamu33G000650), *PaMYB12* (Pamu30G000479), *PaARF4* (Pamu19G001385), and *PaNAC5* (Pamu01G000153) also played important roles in the berberine biosynthesis.

### 3.5. Phylogenetic Tree of the O-Methyltransferase Family

**Figure 5:**
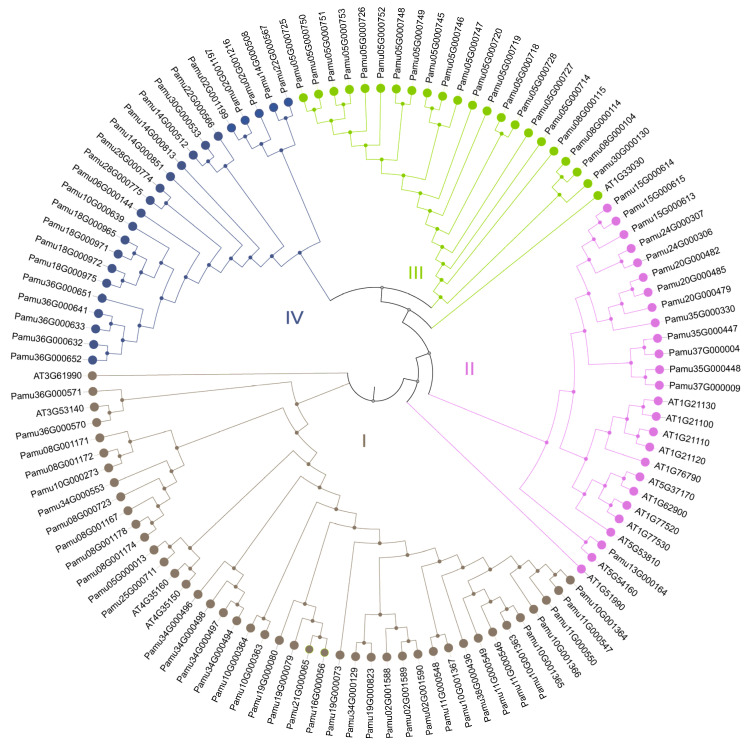
Phylogenetic tree of *OMT*s that encode O-methyltransferases from *Phellodendron amurense* and *Arabidopsis thaliana*. Different colors of branches and nodes represent different subgroups (I–IV).

To elucidate the phylogenetic relationships of *OMT* family members, a phylogenetic tree was constructed using the protein sequences of 97 *OMT*s from *P. amurense* and 17 *OMT*s from *A. thaliana* (Figure 5). The results showed that the 97 *PaOMT*s were clustered into four subgroups (Subgroups I–IV), comprising 23, 21, 14, and 39 *OMT*s, respectively. Subgroup I, the largest subgroup, contained 39 *PaOMT*s and four *AtOMT*s, with the earliest diverging genes being *PaOMT2* (Pamu36G000571 and Pamu36G000570), which showed the closest phylogenetic relationship with AT3G53140 (*AtOMT2*) from *A. thaliana*. Subgroup II contained the largest number of *AtOMT* (12), along with 14 *PaOMT*s. Subgroups II and III were distinguished by AT1G33030 (*AtOMT1*). Subgroup IV consisted of 23 *PaOMT*s, indicating a relatively distant phylogenetic relationship with *AtOMT*s.

## 4. Discussion

The organ-specific activity of plant secondary metabolism is distinctly evident. In *Solanum viarum* Dunal., leaves and roots serve as the main sites of accumulation for bioactive molecules such as glycoalkaloids, phenolics, and flavonoids [28]. In *Meconopsis betonicifolia* Franch., the total alkaloid content is highest in the roots, ranging from 1.86 to 3.28 times that found in the stems, leaves, and flowers [29]. Similarly, in wild *P. amurense* trees aged 20 to 30 years, the contents of berberine and jatrorrhizine in barks were higher than in branches and leaves [14]. In the present study, several key BIAs, such as berberine, jatrorrhizine, and phellodendrine, were significantly upregulated in the stems of *P. amurense* plantlets compared to the leaves. Such organ-specific enrichment of metabolites may participate in the resource allocation in the context of the trade-off between plant growth and defense [30]. Notably, the relative contents of berberine and jatrorrhizine in stems showed a fold change of 41,378.7 and 3612.4, respectively, compared to leaves. These values are substantially higher than previously reported fold changes (1.2–346.2 for berberine and 3.3–14.3 for jatrorrhizine) between bark and branches or leaves [14]. This discrepancy is likely due to the extremely low levels of these two alkaloids in leaves. Therefore, the quantification of specific metabolites is required to validate the magnitude of these differences. Given the commercial availability of berberine and jatrorrhizine standards, such validation is feasible and should be performed in future studies to confirm the tissue-specific accumulation patterns of these alkaloids.

The complex process of plant secondary metabolite biosynthesis is precisely regulated by the expression of multiple genes encoding key enzymes. In *Papaver somniferum* L., transcripts of *PsTyrAT*, which is involved in the synthesis of BIA precursors, are highly enriched in the roots and stems of mature plants; silencing of this gene leads to a nearly 50% reduction in total alkaloid content, underscoring its important role in alkaloid biosynthesis in *P. somniferum* [31]. *MbDDC* exhibits the highest expression level in the roots of *M. betonicifolia*, consistent with the high alkaloid content in this organ; its overexpression results in a significant increase (by 274%) in the total alkaloid content in the roots of *Nicotiana tabacum* L., confirming DDC as a key rate-limiting enzyme in the alkaloid biosynthetic pathway [29]. In contrast, in the present study, multiple key genes involved in upstream aromatic amino acid metabolism in BIA biosynthesis, including *TYDC*, *AspT*, and tyrosine aminotransferase (*TyrAT*), were found to be downregulated in stems of *P. amurense*. Meanwhile, genes primarily involved in downstream steps in BIA biosynthesis, including members of the *OMT*s and *CYP*s, were highly expressed in stems, consistent with the efficient accumulation of berberine in this organ. This organ-specific division is similar to a previous report [32]. Such spatial distribution of the biosynthetic pathway is key to regulating efficient and safe BIA synthesis.

Alkaloid biosynthesis depends not only on the genes encoding key enzymes that directly catalyze biochemical reactions but is also regulated by multiple TFs. In *N. tabacum*, *NtNAC028* and *NtNAC080* form a heterodimer that activates the expression of the core gene *NtLOX3* in jasmonic acid biosynthesis, thereby promoting nicotine biosynthesis and accumulation [33]. The *NnMYC2*-*NnMYB14* module mediates the transmission of jasmonic acid signals to the BIA biosynthesis in *Nelumbo nucifera* Gaertn., while *NnWRKY70a*/*b* directly activates the expression of multiple key genes, collectively participating in the transcriptional regulation of BIA biosynthesis [34]. Similarly, in the present study, TFs such as *PaWRKY12*/*13*, *PaMYB12*, and *PaNAC5* occupy important positions in the regulatory network governing berberine biosynthesis. Notably, *PaBES1* was identified as a core node within this regulatory network. Previous studies have indicated that brassinosteroids (BRs) are one of the major phytohormones regulating stem development in *P. amurense* [15]. Exogenous BR treatment upregulates the expression of *PtTDC*, *Pt6OMT*, and *PtCNMT* in *Pinellia ternata* (Thunb.) Ten. ex Breitenb., resulting in a 90.87% increase in total alkaloid content [35]. However, the role of *PaBES1* in alkaloid biosynthesis remains unclear. Subsequent investigations employing molecular biology approaches are warranted to elucidate its biological function, thereby facilitating the further enhancement of berberine content in *P. amurense* and alleviating the current shortage of its medicinal resources.

The *OMT*s exhibits functional diversity in plants, participating in the methylation of plant-specific metabolites such as lignin, flavonoids, melatonin, and stilbenes [36]. Phylogenetic analysis classified the 97 *PaOMTs* into four subgroups, among which subgroup IV consists entirely of *PaOMT*s, exhibiting a distant relationship with their homologous genes in *A. thaliana*. Notably, *PaOMT7* (Pamu10G000630) and *PaOMT9* (Pamu18G000972), both highly expressed in stems, belong to this subgroup (Figure 3B), suggesting that the formation of subgroup IV may result from a specific gene expansion event. Indeed, such specific expansion has been repeatedly reported in genes involved in BIA biosynthesis and is closely associated with the efficient accumulation of BIA. In *Coptis*, both the *TYDC*s and *NCS*s have undergone significant expansion [37]. The *PaCNMT*s, responsible for key methylation steps in *P. amurense*, have similarly undergone marked expansion driven by LTR retrotransposons [13]. As key enzymes in berberine biosynthesis, the specific expansion of subgroup IV members may imply neo-/sub-functionalization [3,31], potentially enabling their involvement in the specific modification of characteristic secondary metabolites in *P. amurense*, such as berberine. In vitro enzymatic assays should be focused on obtaining the biochemical evidence for the functional divergence of subgroup IV members (e.g., *PaOMT7* and *PaOMT9*). The findings will offer new insights into the evolutionary trajectory of key enzymes involved in alkaloid biosynthesis in medicinal plants.

To alleviate the current supply–demand imbalance of *P. amurense* and to fully realize its medicinal potential, it is essential to develop effective breeding strategies. Polyploid breeding is an important approach for the genetic improvement and development of superior forest tree varieties, contributing significantly to increased biomass and enhanced metabolite content. A marked increase in the contents of major medicinal compounds, including berberine, jatrorrhizine, phellodendrine, and palmatine, was observed in the tetraploids of *P. amurense* obtained by Li et al. [16], along with larger leaves and thicker stems relative to the diploids. Similarly, hexaploid *Cannabis sativa* L. exhibited higher total cannabinoid content, with approximately 65% greater accumulation than diploids [38]. Furthermore, triploid F_1_ hybrids of *Bupleurum chinense* DC. derived from crosses between diploids and tetraploids exhibit significant heterosis, with root dry weight increased by 37.3% and yields of saikosaponins A and D enhanced by approximately 60% compared with their parents [39]. These findings highlight the broad application prospects of polyploid breeding in the genetic improvement of medicinal plants. Looking forward, the creation of additional *P. amurense* polyploids with enhanced medicinal value through asexual and sexual polyploidization, combined with the establishment of efficient tissue culture systems for large-scale propagation, will provide a practical pathway to meet the growing demand for this valuable resource.

## 5. Conclusions

In this study, the stem of *P. amurense* was identified as the predominant site of berberine accumulation, and this metabolic advantage is closely associated with the significantly higher expression of downstream *OMT*s and *CYP*s genes in the pathway of berberine biosynthesis in stems. Phylogenetic analysis further revealed a specific expansion branch (Subgroup IV) within the *OMT* family, in which multiple members were highly expressed in stems. Additionally, *PaBES1* was identified as a key regulatory node in berberine biosynthesis. Collectively, this research provides a scientific basis for the molecular breeding of *P. amurense* germplasm with enhanced medicinal value and metabolic engineering.

## Data Availability

The original data presented in the study are openly available in the Genome Sequence Archive under accession number CRA041653 at https://ngdc.cncb.ac.cn/gsa (accessed on 19 April 2026).

## Supplementary Materials

The following supporting information can be downloaded at: https://www.mdpi.com/article/10.3390/cimb48050464/s1.
