# Supplementary material for: Integrated Transcriptome and Metabolome Analysis Reveals Differential Berberine Biosynthesis in Leaves and Stems of Phellodendron amurense Rupr. Plantlets
Source: Curr Issues Mol Biol. 2026 Apr 29;48(5):464. doi: 10.3390/cimb48050464 (PMC13206598; doi:10.3390/cimb48050464)
Supplement: Supplementary file 1 [file cimb-48-00464-s001.zip › Table S1.pdf]

Table S1. Sequences of primers used in RT-qPCR.

Gene	Forward primer (5′–3′)	Reverse primer (5′–3′)
OMT9	TAGGCTCTACAGTCTTGCTCCT	CTGGAGTCAGTGCCAAGGTAAT
CAO	ATGGGCACTTTGTTAAATGGGC	AAATACCATGCATCAGTGGGGT
RISP	TTCCAAAACGTTGTTCGAGCTC	AACAACATTGAGAAGAAGCGGC
FAD3	TGCTCAAGGAACCATCTTC	TGTCCCACAATGCTGTTTAG
PAL	CTGATAGTTATGGCGTTACCAC	CTTGTTGTTGAGTGAGGCAG
WOX4	ATGCGAACACCGAATGAG	TGGGACAATGGCTAAGACC
IAA16	AAGAAAGTGATAAGCCCAGC	ATCCTTCATTCCTTGTGACC
ADF5	GTCACCGTTGATAAGGTTGG	ATTCTTGATGCTGTCGGG
SCR	CTCAATGGTAGGTTTCCCAG	TCATCAAGTTTGTGGCTCC
SUS1	TTGGAAACTACAGTGATGGC	TCGGAATCTGGATACTTCG
18S rRNA	GTGTTGCTTACCCACGAAA	AAGGGCACAAGGCGGAT
