# Supplementary material for: Integrated Transcriptome and Metabolome Analysis Reveals Differential Berberine Biosynthesis in Leaves and Stems of Phellodendron amurense Rupr. Plantlets
Source: Curr Issues Mol Biol. 2026 Apr 29;48(5):464. doi: 10.3390/cimb48050464 (PMC13206598; doi:10.3390/cimb48050464)
Supplement: Supplementary file 1 [file cimb-48-00464-s001.zip › Table S2.pdf]

Table S2. Relative expression level of target genes in RT-qPCR.

Target genes	Relative expression level
18S rRNA	1
ADF5	9.0169
CuAO	0.7915
FAD3	0.0504
IAA16	6.2058
OMT9	3.4114
PAL	0.4865
RISP	1.0122
SCR	10.3379
SUS1	10.5598
WOX4	19.8013
